# Locomotor Ataxia in the Horse

**Published:** 1891-07

**Authors:** Louis Magnin

**Affiliations:** Veterinarian.


### LOCOMOTOR ATAXIA IN THE HORSE.

By Louis Magnin, Veterinarian.

11th Cuirassiers, France.

(*Translated.*)

There are diseases of the spinal cord like those of the eye; they are still but little understood by the veterinarian. A large part of the diseases of the eye, which are subject to relapses or which give rise to sympathetic troubles of the other eye, are generally confounded with periodic ophthalmia; so, in diseases of the spinal cord, a certain number of morbid enteties are attributed to three or four diseases with which they have no relation, either from an anatomo-pathological point of view or from their character; for example, take the vulgar strain of the back, which, according to M. Trasbot, show often, for alterations, a sclerosis of the spinal cord.

The first notice of locomotor ataxia in the horse, is by Messrs. Weber and Barrier; it is very complete (*Recueil* 1884). The 13th Volume of the *Recueil de Memoires et Observattons sur la médecine vétérinaire militaire* contains two others. One, from Principal Veterinarian Foucher, resembles very much the preceding case; the other, reported by M. Pernet, Veterinarian 25th Artillery, (France), differs in the remarkable peculiarity, that the disease was caused by a lightening stroke, and was cured in a comparatively short time.

I have to report another case. On March 20, 1889, a light-grey gelding, nine years, belonging to the 7th Cuirassiers, came into the infirmary for colic, which it had frequently had before. This horse had always been difficult to mount; before the rider was seated, it would kick vigorously, which I believe indicated a certain amount of pain in the lumbar region.

For two days, the colics were dull; the animal laid down and got up occasionally, but at long intervals. At the end of three or four days, the disease was no better, the animal was dull and dejected, the mucous membranes were slightly congested and yellowish. A pulse of 65, was strong and full. The loins were nearly insensible. In the stable the patient stood with its back arched and its legs drawn together. In trying to move it, its gait was unsteady and difficult; keeping the same position, as when at rest, it walked with short steps, but the hind legs were lifted high, like those of a horse with stringhalt, but less brusquely and jerky. The horse stretched frequently to stale; but was only able to void a very small quantity each time.

Trouble of the kidneys or of the bladder were suspected. Rectal examination did not confirm this diagnosis and the urine was found normal, no trace of albumen or casts. The general condition improved for a few days, when a relapse followed, colics for a short time. Again an improvement, the organic functions became regular, except that of locomotion.

The gait was peculiar, and could not be attributed to a nephrito-cystitis. The front legs had normal play. The movement of the hind legs was peculiar and abnormal. They were advanced under the body, which was arched; they were lifted by a quick flexion of the hock, and then carried forward by an energetic jerk, like the action of a *stepper*, they rested in the air an appreciable instant and then fell heavily to the ground.

The movements were not regular, or coördinated—they were made as if the horse did not know how to walk; he threw, rather than carried the legs forward. There was at times a sudden flexion of the hind leg at rest, the horse stumbled, and nearly fell; in walking the hind quarters swung from side to side.

If taken in a circle the difficulty of movement was greater. Blinders rendered the gait still more unsteady.

The loins showed a greater sensibility than normal on pressure, the hocks flened and the hind quarters dropped, and only returned slowly to their natural position. The irregularity of gait became greater. On April 8th, the rapid flexion of the hocks and the arching of the back were less noticeable than at first, but the incoördination of movement had extended to all four legs. The hind legs were worse and made a veritable zig-zag; the hind legs struck each other and struck the foie-legs. Apparently unconscious flexion of the legs, at rest, or just when brought to the ground took place, and the animal frequently tumbled. The trouble became slightly better with exercise, but a trot was impossible.

If the horse was blindfolded, he seemed nailed to the ground; he could only have moved with the greatest difficulty and threatened to fall at each step. The trouble was worse in the right diagonal biped, and the animal always fell on the left side.

In drinking, after several swallows, the legs were drawn under, as in colic; he wanted to lie down and stretch the head on the ground, with a prolonged moan. After a few minutes of rest, he returned to drink. This was repeated for several days, each time he drank slop feed, but never with dry feed.

Erections were frequent, he reared and imitated the act of copulation; once, he tried to cover a mare near him; while venereal desire may occur with gelding, it had not been a habit with the patient.

About April 20th, the disease became stationary, and a few days later there even seemed to be an improvement. On exercise the shoes were frequently pulled off. The improvement was, however, only relative, it was simply an intermission.

The general condition was always good.

There was never cutaneous anaesthesia, or hyperæsthesia. The head was carried high as in the case observedby Weber and Barrier. Sight and hearing were perfect, and seeing him at rest, one would not suppose him ill. The organic functions were regular; pulse 46, temp. 38.2° C., resp. calm, but slightly acelerated on exercise; defecation and micturition were natural.

With little chance of efficacious therapeutics, he was condemned June 20. He was however seen later, the incoördination was more marked, he fell frequently, but was sold later to a dealer and disappeared from observation.

***

Although we have not been able to recognize in this case the severe and lightening like pains, so common in man, we do not hesitate to determine this to be a case of locomotor ataxia. From the observations of Weber, Barrier and Foucher, the lesions of locomotor ataxia in the horse are not as *systematic* as in man.

By analogy we are authorized to presume, that this case ought to have alterations of the superior cords of the spinal marrow. The colics at the commencement, which seemed to arise from the urinary apparatus, were analagous to the vesical pains in ataxia in man. The moans after drinking, were analagous to the gastric crises in man. The venereal desire was identical to the erections in progressive locomotor ataxia.

